# Modified French Osteotomy for Humeroradial Synostosis in a Child with Multiple Synostoses Syndrome: A Case Report

**DOI:** 10.5704/MOJ.1907.011

**Published:** 2019-07

**Authors:** H Sahdi, AH Rasit, CS Khoo, A Bojeng, BA Nur-Alyana

**Affiliations:** Department of Orthopaedics, Universiti Malaysia Sarawak, Kota Samarahan, Malaysia; * Department of Paediatrics, Hospital Umum Sarawak, Kuching, Malaysia; ** Department of Radiology, Universiti Malaysia Sarawak, Kota Samarahan, Malaysia; *** Department of Nursing, Universiti Malaysia Sarawak, Kota Samarahan, Malaysia

**Keywords:** humerus, radius, synostosis, ankylosis, osteotomy

## Abstract

Congenital humeroradial synostosis can occur as an isolated clinical entity or as part of a syndrome. Bilateral elbow fixed flexion deformity is very incapacitating and challenging to treat. Here we present the case of a boy with fixed flexion deformity of both elbows due bilateral humeroradial synostosis. Other characteristic features of multiple synostoses syndrome were also present in this child, his elder brother and mother. We elected to improve the position of the right elbow by adapting the modified French osteotomy described by Bellemore *et al*.

## Introduction

Multiple synostoses syndrome is characterised by multiple joint fusion including proximal symphalangism of fingers and toes, humeroradial synostosis, carpal and tarsal coalition, alongside with unusual nasal features and conductive deafness^1^. Bilateral elbow ankylosis is functionally devastating. It poses a great challenge to treatment, as the ideal elbow position and method of elbow reconstruction is still debatable. Literature on the management of bilateral humeroradial synostosis in multiple synostoses syndrome is limited.

## Case Report

A six-year old boy presented with stiff elbows since infancy. He was born full term to non-consanguineous parents. There was no antenatal exposure to teratogens. The child has difficulty in performing activities of daily living. He completes schoolwork at kindergarten in a rather slow and awkward manner due to the rigid elbows. He has been utilising modified cutlery to overcome his difficulties. Apart from hearing difficulty, he is an active child who loves to play soccer.

The child’s 41-year old mother and 8-year old only brother had restricted elbow joint movements and hearing impairment. There were no other family members with similar history of joint stiffness and hearing disorder.

The parents were concerned about his slowness in performing academic tasks in school due to the elbow position. We offered to fuse the right elbow in a more functional position. Taking into consideration the child’s concerns, daily activities, hobbies and preferences, it was decided to realign the patient’s elbow to 90 degrees flexion.

**Fig. 1:**
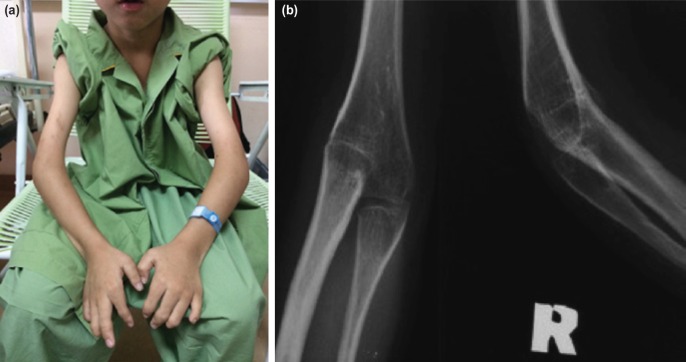
(a) Pre-operative photograph of the patient’s bilateral elbows in fixed flexion of 30 degrees. (b) The pre-operative right elbow radiograph demonstrating humeroradial synostosis with hypoplastic humeroulnar joint.

On examination, the child had striking features of sharp nose and jaw. His elbows were fixed at 30 degrees of flexion (Fig. 1), and midprone forearm position. The fingers and toes appeared to be short with lack of interphalangeal joints bilaterally. Both mother and elder brother also had similar presentations. Examination of the other organ systems was unremarkable.

Plain radiographs of the elbow (Fig. 1) showed ankylosis of the humeroradial joint, with hypoplastic humeroulnar joint. The distal phalanges of ring and little fingers and fourth and fifth toes were absent. The middle phalanges of all fingers and toes were hypoplastic and fused with the proximal phalanges. There was carpal and tarsal coalition. Audiometry showed conductive hearing deafness.

**Fig. 2:**
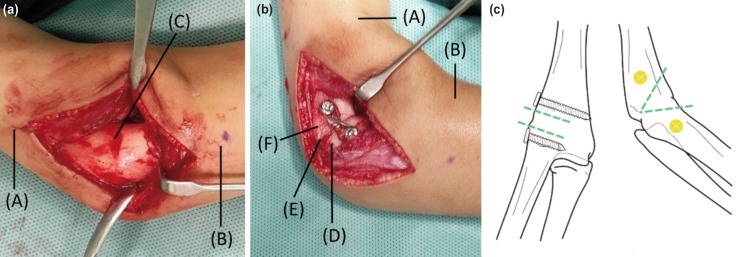
(a and b) Intra-operative photographs and (c) illustration of the right elbow osteotomy. (a) Right elbow photographs showing the humeroradial synostosis before osteotomy and (b) after completion of the French osteotomy and fixation. (c) Illustration of the right elbow showing plane of osteotomy and fixation in antero-posterior position and lateral position. ^*^ (A) Right arm; (B) right forearm; (C) humeroradial synostosis (D) right radius; (E) site of osteotomy; (F) right humerus.

In a supine position, under general anaesthesia, the lateral aspect of the right elbow was approached using the extended Kocher’s incision. The radio-humeral synostosis was accessed through subperiosteal approach (Fig. 2). Two Kirschner wires were placed to mark the plane of osteotomy. Next, two 2.5 mm cortical screws were inserted, with each screw placed proximal and distal from the arthrodesis site, on the midline of the distal humerus lateral surface and the adjacent proximal radius (Fig. 2). Adapting the French osteotomy modification by Bellemore *et al*, anterior wedge osteotomy was performed, leaving the apex at the posterior cortex intact (Fig. 2). Once the desired 90 degrees elbow flexion position was achieved, a figure-of-eight 1.0 mm cerclage wire was tightened around the screw heads (Fig. 2). The elbow was maintained in an above-elbow backslab for six weeks until osseous bridging was achieved.

**Fig. 3:**
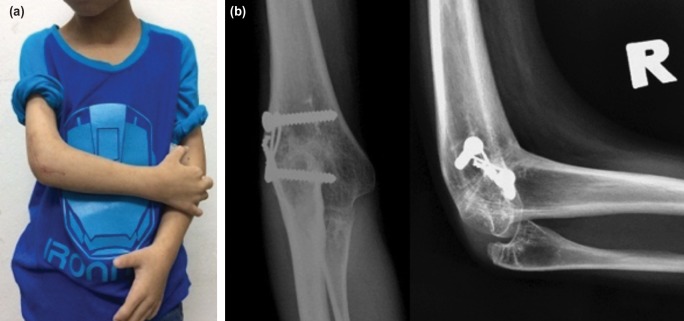
(a) Post-operative photograph and (b) plain radiographs taken one year after surgery showing right elbow position of 90 degrees and union of osteotomy site.

At 12 months post-surgery (Fig. 3), the child was able to eat and drink with the right upper limb without adaptive devices. His writing speed had improved and he managed to complete academic tasks within the stipulated time.

## Discussion

Humeroradial synostosis can occur either as an isolated deformity or as part of a syndrome. Multiple synostoses syndrome is characterised by brachy-symphalangism of the fingers and toes and multiple joint fusions of wrists, feet and elbows. Characteristic facial appearance includes a broad, tubular-shaped or hemicylindrical nose without flared nostrils and thin upper lip. Conductive deafness, hypoplastic or absent middle phalanges are the other features of this syndrome^1^.

Multiple synostoses syndrome can be familial or sporadic. The mode of inheritance in the familial type can be autosomal recessive or dominant. Autosomal recessive multiple synostoses syndrome has no ulnar hypoplasia or distal ulnar malformation. Symmetrical involvement of the limbs is a feature of autosomal dominant subset^1^.

There is no ideal angle or position to fuse the elbow. The patient will face significant functional limitations regardless of selected position. Literatures suggests elbow fusion angle of between 45 to 110 degrees although historically, 90 degrees has been accepted as the best position^2^. The final position of elbow fusion should be determined on an individual basis, considering age, gender, hand dexterity, occupation, functional ability of the ipsilateral shoulder and wrist as well as the contralateral upper limb, functional requirements and patient preference.

Literature on improving elbow position due to humeroradial synostosis in multiple synostoses syndrome is scarce. Kakarala in 20063, utilised the distraction osteogenesis technique with Ilizarov ring fixator system to correct fixed flexion deformity of elbow in a child with bilateral congenital humeroradioulnar synostosis. However, the method requires the patient to accept the inconvenience from the bulky construct, strict compliance with distraction procedure and meticulous pin site dressing.

French^4^ described a method of lateral closing wedge osteotomy using two parallel screws and figure-of-eight wires to correct cubitus varus deformity from malunited supracondylar humerus fractures in 1959. He originally used the posterior approach to the distal humerus and left the periosteum intact medially. Bellemore and associates^5^ modified French’s technique by making a posterolateral incision and kept the medial cortex of the supracondylar humerus intact during the osteotomy. The intact periosteum acted as hinge that provided better control for the reduction of the osteotomy fragments and aided union of the humerus osteotomy. Considering the benefits, we adapted the modified French method by Bellemore by performing an anterior closing wedge osteotomy with intact posterior cortex apex for easier reduction, and fixed the composite using the French method (Fig. 2).

Dome osteotomy, step-cut osteotomy and three-dimensional osteotomy are technically demanding compared to the French method. Fixation solely by K-wires can be complicated with pin tract infection, pin loosening, unsightly scar and loss of fixation^5^. Arthrodesis with plate and screws in this case was not chosen as it required more extensive soft tissue stripping. Furthermore, the atrophic nature of the child’s muscles would cause prominent implant if this method of fixation was utilised. Elbow arthroplasty to reconstruct a functional joint is unsuitable in view of the atrophic bone base and insufficient muscle bulk. Distraction osteogenesis technique requires compliance from patients with regards to long treatment period and pin care. This method has other complications such as pin site problems, nerve palsies, malunion, re-fracture and infection.

In conclusion, fixed flexion of bilateral elbows is very disabling to the patient. Therefore, fusing the elbow in a more functional position is indicated as in this case. We have yet to come across any literature reports of the application of the French osteotomy technique to congenital elbow fusion cases. We have successfully modified the French technique into an anterior closing wedge osteotomy to achieve the desired elbow position in a child with multiple synostoses syndrome.
